# Successful New Generation LAA Closure Device Implantation After Prior Incomplete Surgical LAA Ligation

**DOI:** 10.1016/j.jaccas.2021.06.027

**Published:** 2021-09-15

**Authors:** Hemal Bhatt, Lucy M. Safi, Vladimir Jelnin, Tilak K.R. Pasala, Grant R. Simons

**Affiliations:** a Structural and Congenital Heart Center, Hackensack University Medical Center, Hackensack, New Jersey, USA; b Division of Cardiac Electrophysiology, Hackensack University Medical Center, Hackensack, New Jersey, USA

**Keywords:** fusion imaging, left atrial appendage, percutaneous closure, transesophageal echocardiogram, transseptal puncture, 3D, 3-dimensional, CT, computed tomography, LAA, left atrial appendage, TEE, transesophageal echocardiogram

## Abstract

We present a case of percutaneous closure of a prior incomplete surgical left atrial appendage (LAA) ligation after a failed closure attempt using the first-generation Watchman device. The new generation Watchman FLX device (Boston Scientific) was implanted in this technically and anatomically challenging LAA patient using multimodality fusion imaging. (**Level of Difficulty: Advanced.**)

## Central Illustration

**Figure:**
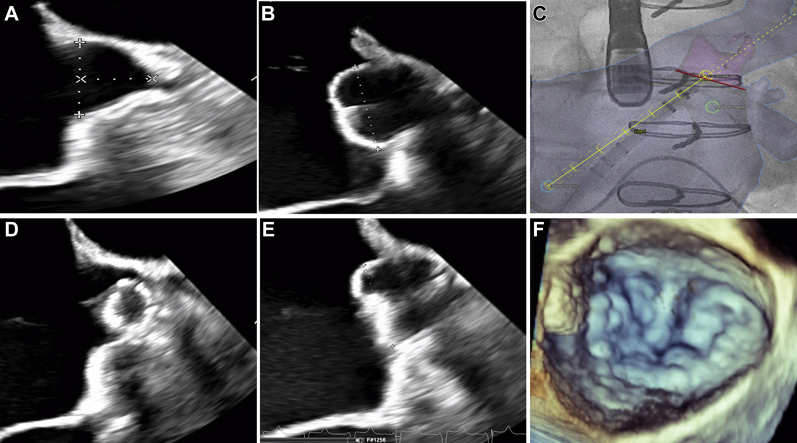

An 82-year-old male (CHA_2_DS_2_-VASc score 4, HAS-BLED score 2), status post prior multiple atrial fibrillation ablations, dual-chamber pacemaker insertion, coronary artery bypass grafting, and surgical left atrial appendage (LAA) ligation (2007) was referred for LAA closure using a new generation LAA closure device. The patient was unsuitable for long-term anticoagulation due to recurrent major oropharyngeal bleeding. Physical examination was unremarkable except for a left infraclavicular pacemaker, and there was no cardiac murmur. Vital signs were 81 beats/min, 141/70 mm Hg blood pressure, 97.9°F body temperature, and 98% oxygen saturation on room air.

**Figure 1:**
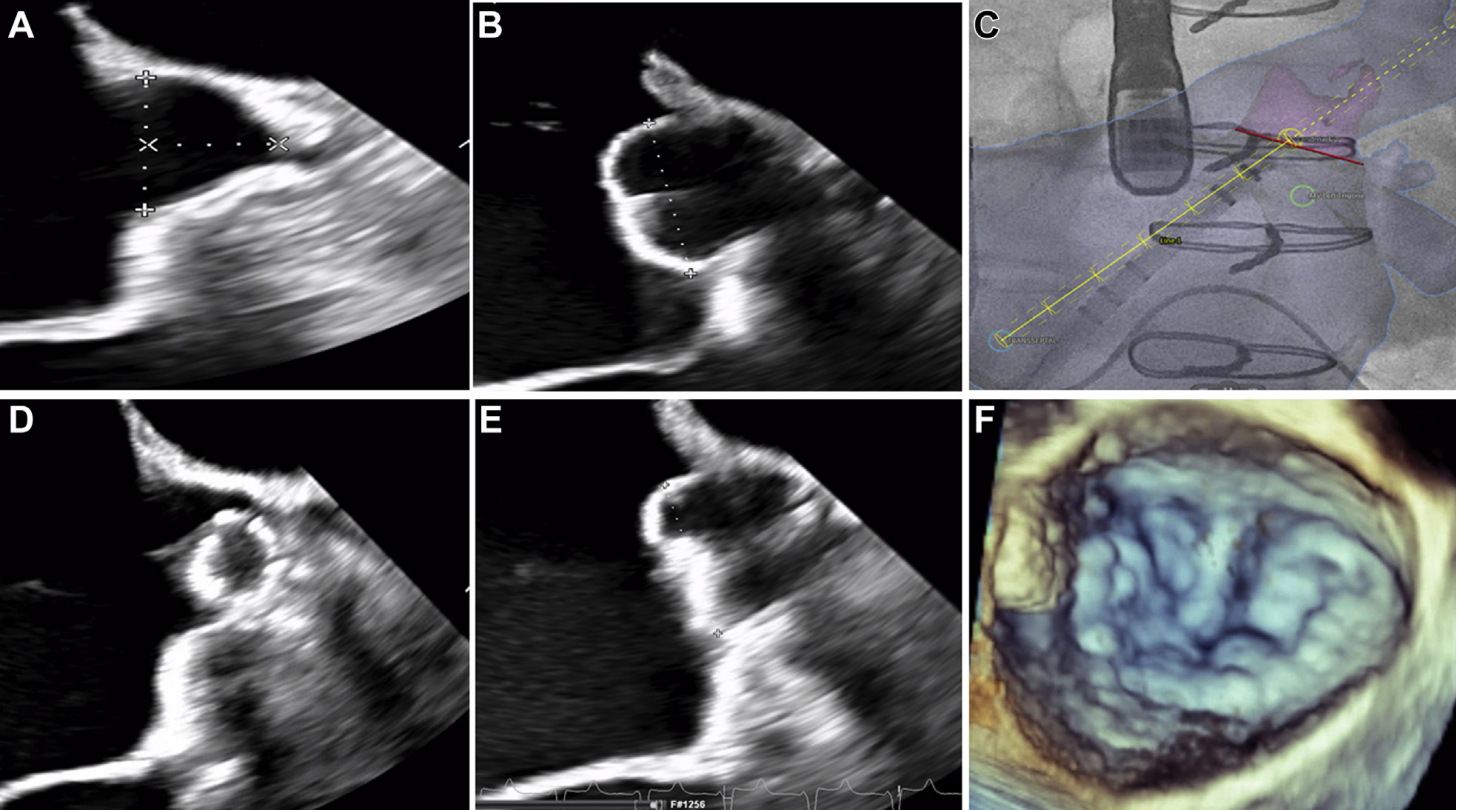
Watchman FLX Implantation **(A)** Left atrial appendage (LAA) stump at 45°. **(B)** Inadequately seated 24-mm new generation LAA closure device. **(C)** Use of fluoroscopy with CT overlay to place 20-mm new generation LAA closure device. **(D)** The device in a Flex ball configuration positioned distally. **(E)** A well-seated device with adequate compression. **(F)** 3-dimensional transesophageal echocardiogram image of the device covering LAA ostium.

Previous transesophageal echocardiogram (TEE) evaluation revealed that the LAA was partially ligated with a residual stump that measured 1.7 cm ostium × 1.3 cm depth (0^o^); 1.8 cm ostium × 1.2 cm depth (45^o^); 1.6 cm ostium × 1.3 cm depth (90^o^); and 1.5 cm ostium × 1.5 cm depth (135^o^) (Figure 1A). This did not communicate with the distal LAA. Because the potential thrombogenicity of such stumps is unknown (1), the patient underwent attempted LAA closure with a 21-mm old generation LAA closure device in July 2020, which was unsuccessful due to inadequate LAA depth. Hence, the patient was referred for LAA closure using a new generation LAA closure device due to its reduced design depth at all diameters. To maximize compression while following the manufacturer’s instructions for use, which specify measuring the ostium from the region of the coronary artery/mitral valve to a point 2 cm from the limbus tip, and using a device providing 10% to 30% compression at the largest measurement, a 24-mm device was initially placed; however, cine angiography and TEE revealed that it was too proximal (Figure 1B). Attempts at implanting the device deeper were unsuccessful due to a shallow usable stump depth. The device was fully recaptured and exchanged for a 20-mm device. Using multimodal fusion imaging, the 20-mm device was positioned (Video 1) and released. The post-release diameter ranged from 16 to 18 mm (10% to 20% compression) and 3-dimensional (3D) TEE revealed a well-seated device (Figures 1C to 1F).

Compared with the previous generation device, the newer-generation Watchman FLX requires half the depth at all widths with an indication for LAA ostia diameters 14 to 31.5 mm. Its closed atraumatic distal end and round Flexball configuration permit safe distal device advancement and deployment in difficult anatomies. The new generation LAA closure device can be redeployed after complete recapture, and it can be advanced after partial recapture (2).

Its reduced depth requirement is also a feature of new generation LAA closure device, which are not currently U.S. Food and Drug Administration (FDA)-approved, such as LAmbre (low-profile fully retrievable device for small LAA), Amulet (disc and lobe configuration for stability at shallow depths) and WaveCrest (proximal positioning without requiring delivery sheath placement into LAA) devices (3). The present case illustrates that the new generation LAA closure device allows for successful deployment in a shallow post-surgical anatomy.

## Funding Support and Author Disclosures

The authors have reported that they have no relationships relevant to the contents of this paper to disclose.

## Appendix

Supplemental Video 1 Fusion Imaging Showing the Final Positioning of the Device
